# Community-Based Harm Reduction Substance Abuse Treatment with Methamphetamine-Using Men Who Have Sex with Men

**DOI:** 10.1007/s11524-014-9870-y

**Published:** 2014-04-18

**Authors:** Adam W. Carrico, Annesa Flentje, Valerie A. Gruber, William J. Woods, Michael V. Discepola, Samantha E. Dilworth, Torsten B. Neilands, Jennifer Jain, Michael D. Siever

**Affiliations:** 1 University of California, San Francisco School of Nursing, 2 Koret Way, N511M, San Francisco, CA 94143 USA; 2 Center for AIDS Prevention Studies, University of California, San Francisco, San Francisco, CA USA; 3 Department of Psychiatry, University of California, San Francisco, San Francisco, CA USA; 4 San Francisco AIDS Foundation, San Francisco, CA USA

**Keywords:** Cognitive-behavioral, Harm reduction, HIV risk, Marijuana, Methamphetamine, Treatment as prevention

## Abstract

Harm reduction approaches endeavor to assist individuals with avoiding the most detrimental consequences of risk taking behaviors, but limited research has documented the outcomes of harm reduction substance abuse treatment. In total, 211 methamphetamine-using men who have sex with men (MSM) enrolled in two outcome studies of substance abuse treatment programs that were implementing an evidence-based, cognitive-behavioral intervention (i.e., the Matrix Model) from a harm reduction perspective. Study 1 (*N* = 123) examined changes in self-reported substance use, Addiction Severity Index (ASI) composite scores, and HIV care indicators over a 12-month follow-up. Study 2 (*N* = 88) assessed changes in substance use, sexual risk taking, and HIV care indicators over a 6-month follow-up. Participants in study 1 reported reductions in cocaine/crack use as well as decreases in the ASI drug and employment composite scores. Among HIV-positive participants in study 1 (*n* = 75), 47 % initiated or consistently utilized anti-retroviral therapy and this was paralleled by significant increases in self-reported undetectable HIV viral load. Study 2 participants reported reductions in methamphetamine use, erectile dysfunction medication use in combination with other substances, and sexual risk-taking behavior while using methamphetamine. Participants in both studies reported concurrent increases in marijuana use. Taken together, these studies are among the first to observe that clients may reduce stimulant use and concomitant sexual risk-taking behavior during harm reduction substance abuse treatment. Randomized controlled trials are needed to examine the differential effectiveness of harm reduction and abstinence-based approaches to substance abuse treatment.

## Introduction

The use of stimulants (i.e., methamphetamine, cocaine, and crack) has negative implications for HIV prevention and care. Among HIV-negative men who have sex with men (MSM), those who use stimulants are more likely to report engaging in sexual risk-taking behavior and are at elevated risk for HIV seroconversion.^1^–^3^ It is also well-established that HIV-positive stimulant users are more likely to experience difficulties with HIV disease management which lead to elevated HIV viral load and potentially faster mortality.^4^–^7^ These difficulties with HIV disease management often co-occur with HIV transmission risk behavior, 8,9 which could contribute to the onward transmission of medication-resistant strains of HIV.^10^,^11^

Behavioral treatments for stimulant dependence are moderately effective.^1^,^12^ Specifically, prior clinical research conducted by Shoptaw and colleagues supports the efficacy and effectiveness of a cognitive-behavioral treatment (i.e., the Matrix Model) that is culturally tailored for MSM.^13^,^14^ One randomized controlled trial (RCT) with methamphetamine-using MSM observed that those randomized to receive a culturally tailored Matrix Model intervention reported greater reductions in unprotected receptive anal intercourse during the first 4 weeks of treatment.^13^ In a second RCT, gay and bisexual men seeking treatment for stimulant or alcohol abuse were randomized to receive this culturally tailored Matrix Model intervention or gay-specific social support therapy.^14^ Secondary analyses indicated that methamphetamine-using participants in the culturally tailored Matrix Model intervention reported greater reductions in methamphetamine use over 1 year than those in gay-specific social support therapy. Although findings support the clinical utility of the Matrix Model with methamphetamine-using MSM, these RCTs focused on testing abstinence-based approaches to treatment.

Harm reduction is a client-centered philosophy that engages clients in the process of behavior change even if they are not motivated to abstain from substance use or refrain from engaging in other risk-taking behaviors.^15^,^16^ In the HIV prevention field, harm reduction interventions such as needle exchange are widely considered to be effective and have been successfully implemented for decades.^17^ There is also some evidence to support the efficacy of other behavioral interventions that are implemented from a harm reduction perspective. One recent RCT with HIV-positive, methamphetamine-using MSM demonstrated that it is possible to promote sexual risk reduction without decreasing methamphetamine use.^18^ The present studies documented the outcomes of methamphetamine-using MSM who were receiving community-based, harm reduction substance abuse treatment in order to determine whether more definitive clinical research is warranted to examine the effectiveness of this approach. The primary outcomes were self-reported stimulant use and Addiction Severity Index (ASI) composite scores. Secondary outcomes included: other substance use, sexual risk taking, anti-retroviral therapy (ART) utilization, and self-reported undetectable HIV viral load. We hypothesized that participants would report decreases in self-reported stimulant use and ASI composite scores as well as concomitant reductions in sexual risk taking and improvements in HIV care indicators.

## Methods

### The Stonewall Project Harm Reduction Treatment Model

The Stonewall Project model translates evidence-based interventions such as the Matrix Model 13,14 into a clinical setting with a harm reduction focus. 19 Harm reduction is a client-centered philosophy which does not assume that all individuals are ready, willing, and able to pursue abstinence as a treatment goal. It also acknowledges that clients often seek out substance abuse treatment services to address risky injection practices and sexual risk-taking behavior, even when they are not interested in changing substance use patterns. Consistent with the harm reduction philosophy, the Stonewall Project model assists clients with pursuing self-identified treatment goals as a means of engaging individuals who might not otherwise initiate or remain in abstinence-based substance abuse treatment. Regarding substance use, clients can choose to pursue abstinence as a treatment goal, but strategies for managing substance use are often a primary focus of treatment. Selected substance use management strategies in the Stonewall Project model include: (1) transitioning to less potent modes of methamphetamine administration (e.g., injecting to smoking, smoking to snorting); (2) promoting self-care strategies while using methamphetamine (e.g., hydration, nutrition); and (3) delivering education about safer injection practices with linkage to needle exchange and access to sterile syringes. The Stonewall Project model also delivers sexual risk-reduction interventions to promote condom use during anal sex as well as seroadaptive behaviors (e.g., serosorting, strategic positioning) for when clients choose not to use condoms.^20^ Clients are encouraged to develop plans for addressing barriers to sexual risk reduction, even in the context of methamphetamine use. As part of the Stonewall Project model, clients receive outpatient treatment that consists of weekly individual counseling, group counseling twice a week, and psychotropic medications where appropriate. Unlike the Matrix Model,^13^,^14^ clients are not asked to provide weekly urine samples to test for recent stimulant use as part of their ongoing treatment. This 1-year outpatient drug treatment program has been implemented for over 15 years by the community-based substance abuse treatment programs that were the focus of the present outcome studies.

### Treatment Outcome Study 1

#### Procedures

Study 1 was conducted with two co-located outpatient programs that were implementing the Stonewall Project model. One of the programs specialized in the treatment of methamphetamine-using MSM. The other served methamphetamine users of any gender or sexual orientation, but only MSM clients were included in the present study. All consecutive admissions to these programs from March 2005 to May 2008 were eligible. At the end of their program intake session, interested clients gave permission for research staff to contact them (i.e., self-selection).

At the baseline assessment, research staff uninvolved with treatment services completed the informed consent process and administered the ASI at the substance abuse treatment site. At 6 and 12 months, the follow-up ASI was administered at the substance abuse treatment site. In total, 132 methamphetamine-using MSM enrolled in study 1, but nine were removed because they were also enrolled in study 2. Of the 123 unduplicated participants, 107 (87 %) and 98 (80 %) completed follow-up assessments at 6 and 12 months, respectively. In total, 112 participants (91 %) completed at least one follow-up assessment over the 12-month period. For their time and travel expenses, participants received US$10 at the baseline and 6-month assessments as well as US$20 at the 12-month assessment. All participants signed a separate authorization allowing research staff to extract data regarding treatment utilization from clinical billing records. Study 1 procedures were approved by the University of California, San Francisco Committee on Human Research.

#### Measures

Demographic information including items assessing sexual orientation and gender identity was obtained from a modified demographics form for the ASI. Among HIV-positive persons, self-reported ART utilization and undetectable viral load were assessed via questionnaire. The ASI was administered at each study visit. Responses were entered into ASI Drug Evaluation Network System (DENS) software, which calculated composite scores.^21^ As part of the ASI, participants reported the number of days that they had used specific substances in the past 30 days. Substances examined in this study included: methamphetamine, cocaine/crack, marijuana, and drinking to intoxication.

### Treatment Outcome Study 2

#### Procedures

Methamphetamine-using MSM were recruited from the outpatient substance abuse treatment program in study 1 that is implementing the Stonewall Project model exclusively with MSM from July 2010 to June 2012.^22^ Clients receiving treatment at the Stonewall Project were eligible to enroll in study 2 up to: (1) 60 days after treatment initiation; or (2) 60 days following re-initiation after more than 30 days out of treatment (NCT 01129401). Clients who were interested in participating completed a consent to contact form with their Stonewall Project counselor (i.e., self-selection) that included permission to verify eligibility using treatment records.

After providing informed consent at the baseline assessment visit, participants completed an assessment administered by a research assistant at the substance abuse treatment site. This assessment included measures of self-reported substance use and sexual risk taking that were administered using audio computer-assisted self-interviewing (ACASI). Prior research has demonstrated that ACASI enhances the reliability of self-report measures for substance use and sex risk.^23^,^24^ This assessment was re-administered at 3 and 6 months follow-up at the substance abuse treatment site. Participants also provided a urine sample for on-site toxicology screening for methamphetamine and cocaine metabolites at each study assessment (Redicup®; Redwood Toxicology Laboratory; Santa Rosa, CA). All study assessments were administered by a research assistant who did not have a clinical role in the substance abuse treatment program.

Of the 88 participants enrolled in this treatment outcome study, 81 (92 %) and 79 (90 %) completed follow-up assessments at 3 and 6 months, respectively. In total, 85 participants (96 %) completed at least one follow-up assessment over the 6-month period. At each study visit, participants were reimbursed with a US$50 pre-loaded debit card for their time and travel expenses. All participants signed a separate authorization allowing research staff to extract data regarding treatment utilization from the clinical records. Study 2 procedures were approved by the University of California, San Francisco Committee on Human Research.

#### Measures

Demographic characteristics assessed by questionnaire included: age, ethnicity, education, income, sexual orientation, gender identity, and HIV status. Among HIV-positive persons, self-reported ART utilization and undetectable HIV viral load were assessed at each study visit. Consistent with study 1, participants reported the number of days they used specific substances in the past 30 days. Substances examined in this study included: methamphetamine, cocaine/crack, club drugs (i.e., ecstasy, ketamine, or GHB), marijuana, binge drinking (i.e., six or more drinks on one occasion), and erectile dysfunction (ED) medications in combination with other substances (i.e., while “partying”). Participants also reported the longest number of days in a row they used cocaine/crack or methamphetamine in the past 30 days. Informed by previous research with methamphetamine users, 25 participants who reported using any of these stimulants two or more days in a row were classified as engaging in binge use (1) and compared to those who reported using 1 day at a time or no use in the past 30 days (0). Participants who provided a urine sample that was reactive for cocaine or methamphetamine metabolites (1) were compared to those who provided a urine sample that was not positive for recent stimulant use (0).

Participants reported the number of anal sex partners in the past 3 months, stratified by whether or not they were feeling the effects of methamphetamine during sexual intercourse. For HIV-negative MSM, sexual risk-taking behavior was operationalized as engaging in unprotected anal intercourse, irrespective of the serostatus of the sexual partner(s). For HIV-positive MSM, sexual risk-taking behavior was operationalized as unprotected anal intercourse with HIV-negative or unknown serostatus partners. Estimates of any sexual risk-taking behavior were calculated separately for receptive and insertive anal sex as a function of whether participants were using methamphetamine to yield four separate indicators.

### Statistical Analyses

Inferential analyses examining unadjusted change over time for each dependent variable were performed with generalized estimating equations (GEE) in Stata using the binomial distribution and logit link for binary dependent variables (e.g., ART, self-reported undetectable HIV viral load), the multinomial distribution and cumulative logit link for ordinal categorical dependent variables (i.e., ASI Alcohol and Medical composite scores), the negative binomial distribution and log link for count dependent variables (e.g., number of methamphetamine days, number of anal sex partners), and the normal distribution and identity link for continuous dependent variables (e.g. ASI Drug and Employment composite scores). As recommended by Diggle, 26 the unstructured covariance structure was used for binary, count, and continuous outcomes. For the ordinal categorical dependent variables, the independent covariance structure was used. Multiple imputation was employed to handle missing data for any models with incomplete data due to both participant non-response (≤18 % in study 1, <2 % in study 2) and loss to-follow-up (≤20 % in study 1, ≤10 % in study 2). Thus, the total sample size is the same (*N* = 123 for study 1, *N* = 88 for study 2) in each reported analysis.

For binary outcomes, the odds ratio (OR) per unit change in the independent variable is reported. For count outcomes, we report the incidence rate ratio (IRR) as well as the percent change in the expected number of the outcome (Δ expected = 100 × (*e* ^*B*^ − 1)). For continuous outcomes, the raw change in the outcome per unit change in the independent variable (*B*) is reported. Observed effect sizes were determined using Cohen’s *d* for continuous and count dependent variables, Cohen’s *h* for binary dependent variables, and the Uncertainty Coefficient (c|r) for ordinal dependent variables.^27^–^29^

Dose–response analyses were performed only for dependent variables that exhibited significant change over time. To improve imputations by allowing information contained in other non-missing variables to inform missing values, all dependent variables in the dose–response analyses were used together to create 50 imputed datasets. Three different dose variables were examined individually as correlates of change over time for each significant dependent variable: number of individual counseling sessions, number of group counseling sessions, and number of psychiatric sessions. The linearity assumption, assessed via the cumulative sums of residuals method, 30 was not violated in any of the models. To assess the associations of dose variables with outcomes over time, the interaction of dose and time was considered in a model including both main effects. In the two instances where the dose-time interaction was found significant, the associations for dose were calculated at the final follow-up visit for that study.

## Results

### Demographics and Health Status

Overall, participants in study 1 and study 2 were predominantly gay-identified, Caucasian, middle-aged, and HIV-positive. As shown in Table 1, participants in study 1 were younger, less likely to be prescribed ART at baseline, and less likely to report undetectable HIV viral load at baseline. In study 1, 27 % of participants reported consistently utilizing ART while 20 % reported initiating and remaining on ART during the 12-month follow-up period. In study 2, 75 % of participants reported consistently utilizing ART while 2 % reported initiating and remaining on ART following during the 6-month follow-up period.

**TABLE 1:** Comparison of demographic characteristics and health status at baseline

	Study 1	Study 2	*p* value
	(*N* = 123)	(*N* = 88)	
	*N* (%)	*N* (%)	
Gender	–	–	0.34
Male	121 (98.4)	88 (100.0)	
Transgender (female to male)	2 (1.6)	0 (0.0)	
Ethnicity	–	–	0.42
African American	9 (7.5)	10 (11.4)	
Hispanic/Latino	26 (21.7)	12 (13.6)	
Caucasian	79 (65.8)	59 (67.1)	
Asian/Pacific Islander	2 (1.7)	2 (2.3)	
American Indian/Alaskan	1 (0.8)	0 (0.0)	
Multicultural	3 (2.5)	5 (5.7)	
Sexual orientation	–	–	0.11
Gay	106 (89.1)	85 (96.6)	
Bisexual	10 (8.4)	2 (2.3)	
Straight	1 (0.8)	1 (1.1)	
Unsure	2 (1.7)	0 (0.0)	
Education	–	–	0.10
Less than high school	11 (9.2)	3 (3.4)	
High school graduate	19 (16.0)	13 (14.8)	
Trade school or some college	44 (37.0)	34 (38.6)	
College graduate	24 (20.2)	29 (33.0)	
Graduate degree	21 (17.7)	9 (10.2)	
Employed	34 (29.3)	25 (28.7)	0.93
Currently homeless	16 (13.2)	9 (10.2)	0.51
Ever incarcerated	34 (28.8)	24 (27.3)	0.81
HIV-positive	75 (63.6)	58 (65.9)	0.73
Currently prescribed anti-retroviral therapy	33 (44.0)	50 (86.2)	< 0.0001
Self-reported undetectable HIV viral load	14 (18.6)	35 (60.3)	< 0.0001
	M (SD)	M (SD)	
Age	40.7 (7.5)	43.3 (9.0)	0.02
Years since HIV diagnosis	–	10.8 (7.8)	–
Self-reported T-helper (CD4^+^) count	444 (239)	527 (295)	0.11

### Treatment Outcome Study 1 Analyses

As shown in Table 2, there were significant reductions in cocaine/crack use days between 6 and 12 months (IRR = 0.54; 95 % confidence Interval [CI] = 0.32, 0.91; *p* < 0.05; Δ expected = −46.3 %). Participants also reported concurrent increases in marijuana use days over the 12-month follow-up (IRR = 1.46; 95 % CI = 1.02, 2.09; *p* < 0.05; Δ expected = 45.7 %). There were reductions in the ASI Drug composite score over the 12-month follow-up (*B* = −0.03; 95 % CI = −0.05, −0.01; *p* < 0.01), indicating a decrease in drug use severity. There were also decreases in the ASI Employment composite score over the 12-month follow-up (*B* = −0.04; 95 % CI = −0.08, −0.01; *p* < 0.05), representing an improvement in employment outcomes. Lastly, more HIV-positive participants reported an undetectable viral load over the 12-month follow-up (OR = 2.23; 95 % CI = 1.12, 4.41; *p* < 0.05).

**TABLE 2:** Changes in outcomes over time for study 1 (*N* = 123)

	Baseline	6 months	12 months	Effect size	
	*M* (SD)	*M* (SD)	*M* (SD)	Cohen’s *d*	
Methamphetamine-use days	4.85 (8.06)	4.57 (7.39)	4.92 (7.67)	0.01	
Cocaine/crack-use days	1.67 (5.59)	2.05 (6.72)	1.06 (4.06)	−0.12	*
Marijuana-use days	3.55 (7.72)	4.12 (8.38)	5.87 (9.95)	0.26	*
Drinking-to-intoxication days	1.22 (3.32)	0.94 (3.41)	1.18 (2.96)	−0.01	
ASI employment score	0.65 (0.31)	0.61 (0.31)	0.58 (0.29)	−0.23	*
ASI drug score	0.19 (0.10)	0.16 (0.12)	0.17 (0.11)	−0.19	**
ASI family/social score	0.20 (0.17)	0.16 (0.18)	0.17 (0.17)	−0.18	
ASI psychiatric score	0.36 (0.21)	0.37 (0.21)	0.35 (0.22)	−0.05	
	*N* (%)	*N* (%)	*N* (%)	Cohen’s *h*	
ASI legal score	31 (26.7)	27 (26.0)	22 (22.9)	−0.09	
On ART (self-report)	33 (46.5)	40 (58.0)	29 (47.5)	0.02	
Undetectable HIV viral load (self-report)	14 (20.0)	24 (42.1)	22 (37.3)	0.38	**
	*N* (%)	*N* (%)	*N* (%)	UC c|r	
ASI medical score				0.0042	
Zero	54 (48.2)	49 (46.8)	44 (47.3)		
<=0.5†	30 (26.8)	31 (29.5)	19 (20.4)		
>0.5	28 (25.0)	25 (23.8)	30 (32.3)		
ASI alcohol score				0.0013	
Zero	45 (39.5)	42 (40.0)	38 (40.4)		
<=0.128^a^	36 (31.6)	34 (32.4)	33 (35.1)		
>0.128	33 (29.0)	29 (27.6)	23 (24.5)		

*ART* anti-retroviral therapy, *UC c|r* uncertainty coefficient

*p < 0.05; **p < 0.01

^a^Pseudo tertiles: 0 as the bottom category, and the second two categories bisect the remaining data

For the crack/cocaine and marijuana use days as well as the ASI Drug composite score, there were no significant dose–response associations. A greater number of individual counseling sessions (*B* = −0.001; 95 % CI = −0.002, −0.001; *p* < 0.01) and psychiatric sessions (*B* = −0.006; 95 % CI = −0.01, −0.003; *p* < 0.01) attended were associated with reductions in the ASI Employment composite score, which is indicative of improved employment outcomes. For self-reported undetectable HIV viral load, there was a significant effect for the dose × time interaction for individual counseling sessions, but the change in the outcome over time remains significant (OR = 3.47; 95 % CI = 1.37, 8.79; *p* < 0.01) with the inclusion of the interaction. The dose effect of the number of individual counseling sessions was not significant at 12 months. These results using imputed data from study 1 were consistent with sensitivity analyses using data that were not imputed.

### Treatment Outcome Study 2 Analyses

As shown in Table 3, participants reported reductions in days of methamphetamine use over the 6-month follow-up (IRR = 0.71; 95 % CI = 0.52, 0.96; *p* < 0.05; Δ expected = −29.4 %), increases in marijuana use through the 3-month follow-up (IRR = 1.36; 95 % CI = 1.02, 1.82; *p* < 0.05; Δ expected = 36.1 %), and reductions in ED medication use in combination with other substances over the 6-month follow-up (IRR = 0.37; 95 % CI = 0.21, 0.66; *p* < 0.01; Δ expected = −63.1 %). Participants also reported concurrent reductions in the number of anal sex partners while using methamphetamine over the 6-month follow-up (IRR = 0.45; 95 % CI = 0.27, 0.73; *p* < 0.01; Δ expected = −55.1 %) as well as decreased odds of any receptive sexual risk taking while using methamphetamine over the 6-month follow-up (OR = 0.53; 95 % CI = 0.30, 0.94; *p* < 0.05). Although the percentage of participants reporting undetectable viral load increased over time, this effect was not statistically significant at 6 months (OR = 1.60; 95 % CI = 0.89, 2.86; *p* > 0.05). There was one significant dose–response association amongst the outcomes that exhibited change over time. Each group counseling session attended was associated with a 2.4 % reduction in days of methamphetamine use over the 6-month follow-up (IRR = 0.98; 95 % CI = 0.96, 0.99; *p* < 0.01; Δ expected = −2.37 %). These results using imputed data from study 2 were consistent with sensitivity analyses using data that were not imputed.

**TABLE 3:** Changes in outcomes over time for study 2 (*N* = 88)

	Baseline	3 months	6 months	Effect size	
	*M* (SD)	*M* (SD)	*M* (SD)	Cohen’s *d*	
Meth-use days	5.23 (7.82)	5.06 (8.28)	3.57 (6.11)	−0.24	*
Cocaine/crack-use days	0.77 (2.81)	0.58 (2.55)	0.58 (2.02)	−0.08	
Club-drug-use days	1.41 (4.41)	1.25 (4.37)	0.81 (2.71)	−0.16	
Marijuana-use days	4.59 (8.69)	6.14 (10.29)	5.35 (9.77)	0.08	*
Binge-drinking days	1.17 (2.96)	1.04 (2.84)	0.78 (1.92)	−0.16	
ED-medication-use-while-“Partying” days	0.80 (2.50)	0.80 (3.56)	0.29 (0.64)	−0.28	*
Number of anal sex partners on meth	5.16 (10.33)	3.24 (9.83)	2.32 (6.66)	−0.33	**
Number of anal sex partners not on meth	2.06 (5.31)	1.58 (4.02)	1.56 (4.03)	−0.04	
	*N* (%)	*N* (%)	*N* (%)	Cohen’s *h*	
Any binge stimulant use	43 (49)	35 (43)	34 (43)	−0.13	
Tox+ for stimulants	27 (32)	31 (40)	22 (32)	0.00	
Any risky anal sex	35 (41)	29 (37)	29 (37)	−0.15	
Any risky RAS on meth	23 (26)	13 (17)	13 (17)	−0.24	*
Any risky RAS not on meth	7 (8)	6 (8)	9 (12)	0.11	
Any risky IAS on meth	17 (20)	12 (15)	9 (12)	−0.22	
Any risky IAS not on meth	9 (10)	6 (8)	6 (8)	−0.09	
On ART	50 (86)	41 (79)	42 (82)	−0.10	
Undetectable viral load (self-report)	35 (60)	35 (67)	37 (72)	0.26	

*Meth* methamphetamine, *ED* erectile dysfunction, *Tox*+ reactive urine sample, *RAS* receptive anal sex, *IAS* insertive anal sex, *ART* anti-retroviral therapy

*p < 0.05, **p < 0.01

## Discussion

To our knowledge, these studies are among the first to document the outcomes of community-based, outpatient substance abuse treatment that is being delivered from a harm reduction perspective. In study 1, participants reported decreases in cocaine/crack use as well as reductions in drug use severity and improvements in employment status. Because harm reduction is a client-centered approach that encourages individuals to identify and pursue their own treatment goals, it does not require abstinence. Although we observed increases in marijuana use, participants in study 1 also showed improvements in the drug use severity composite score of the ASI which indexes polysubstance use, drug-related problems, and perceived need for treatment. Improvements in employment status provide further support for enhanced functioning in a key life domain, which for many participants was in the context of ongoing substance use. Participants in study 2 reported reductions in the frequency of methamphetamine use and concurrent increases in marijuana use, but no measure of drug use severity was administered. Taken together, these results provide preliminary support for the potential benefits of harm reduction treatment for assisting individuals with reducing stimulant use and minimizing its potential negative consequences. Further clinical research in this area is warranted.

Substance abuse treatment can support HIV/AIDS prevention efforts, 31,32 and study 2 observed reductions in HIV-related risk behaviors. Participants reported reductions in the use of ED medications in combination with other substances. One common side effect of stimulants such as methamphetamine is impaired erectile functioning, 33 and men often use ED medications in combination with methamphetamine to achieve an erection, prolong sexual activity, or enhance sexual pleasure.^34^ Because it is an independent risk factor for HIV seroconversion, 3,35 reductions in the use of ED medications in combination with other substances may have important clinical implications. Consistent with prior clinical research, 13,14,18 participants also reported decreases in the number of anal sex partners while using methamphetamine as well as decreased odds of engaging in any risky receptive anal sex while using methamphetamine. Although more definitive clinical research is clearly needed, findings highlight the potential benefits of harm reduction substance abuse treatment for decreasing the co-occurrence of methamphetamine use and sexual risk taking among MSM.

Initiating and remaining on ART at higher T-helper (CD4^+^) counts can lead to better health outcomes among HIV-positive persons as well as decrease onward HIV transmission rates.^36^ In San Francisco, universal access to ART irrespective of CD4^+^ count was implemented in 2010, which led to reductions in HIV viral load among those enrolled in HIV medical care.^37^ Consistent with these policy changes, the baseline prevalence of ART utilization in study 2 (2010–2012) versus study 1 (2005–2008) was significantly higher (86 % vs. 47 %). Participants in study 1 reported increases in self-reported undetectable HIV viral load, which may be due in large part to the fact that one-fifth of those enrolled in this study initiated and remained on ART during the 12-month follow-up period. In contrast, more than half of HIV-positive participants enrolled in study 2 (60 %) reported undetectable HIV viral load at baseline. This restricted range combined with the fact that three-fourths of HIV-positive participants consistently remained on ART during the 6-month follow-up may account for the absence of a statistically significant increase in self-reported undetectable HIV viral load. The potential for substance abuse treatment to optimize the effectiveness of HIV treatment as prevention remains an important area for further clinical research.^38^

Findings from the present studies must be interpreted in context of some important limitations. Because these studies documented the outcomes of community-based substance abuse treatment programs, it was not ethical to withhold treatment even temporarily in a wait-list control design. Although reductions in stimulant use and concomitant sexual risk taking are largely consistent with prior randomized controlled trials of the Matrix Model with this population,^13^,^14^ it is not possible to determine whether observed changes are attributable to regression to the mean without a comparison condition. It is also noteworthy that reductions in the frequency of methamphetamine use in study 2 were observed in the absence of concurrent decreases in the proportion of participants providing a urine sample that was reactive for stimulants. Urine biomarkers verify self-reported abstinence, but they do not index cumulative exposure to stimulants over time. Further research is needed to examine quantitative biomarkers (e.g., hair toxicology) that may better reflect frequency and quantity of stimulant use over longer periods. Although there is some limited support for the reliability and validity of self-reported undetectable HIV viral load, 39 future research should also collect peripheral venous blood samples to measure changes in HIV disease markers.

Harm reduction policies and programs are being implemented around the globe to better meet the needs of those who are not ready, willing, or able to abstain from substance use or refrain from engaging in other risk-taking behaviors.^40^ Randomized controlled trials are needed to examine the differential effectiveness of harm reduction and abstinence-based approaches to substance abuse treatment. At the same time, these studies are among the first to observe that clients may reduce stimulant use and concomitant sexual risk-taking behavior during harm reduction substance abuse treatment.
